# Cordycepin Suppresses Expression of Diabetes Regulating Genes by Inhibition of Lipopolysaccharide-induced Inflammation in Macrophages

**DOI:** 10.4110/in.2009.9.3.98

**Published:** 2009-06-30

**Authors:** Seulmee Shin, Sungwon Lee, Jeonghak Kwon, Sunhee Moon, Seungjeong Lee, Chong-Kil Lee, Kyunghae Cho, Nam-Joo Ha, Kyungjae Kim

**Affiliations:** 1 College of Pharmacy, Sahmyook University, Seoul, Korea.; 2 College of Pharmacy, Chungbuk University, Cheongju, Korea.; 3 Department of Biology, Seoul Women's University, Seoul, Korea.

**Keywords:** cordycepin, type 2 diabetes, pro-inflammatory cytokines, immunomodulator

## Abstract

**Background:**

It has been recently noticed that type 2 diabetes (T2D), one of the most common metabolic diseases, causes a chronic low-grade inflammation and activation of the innate immune system that are closely involved in the pathogenesis of T2D. *Cordyceps militaris*, a traditional medicinal mushroom, produces a component compound, cordycepin (3'-deoxyadenosine). Cordycepin has been known to have many pharmacological activities including immunological stimulating, anti-cancer, and anti-infection activities. The molecular mechanisms of cordycepin in T2D are not clear. In the present study, we tested the role of cordycepin on the anti-diabetic effect and anti-inflammatory cascades in LPS-stimulated RAW 264.7 cells.

**Methods:**

We confirmed the levels of diabetes regulating genes mRNA and protein of cytokines through RT-PCR and western blot analysis and followed by FACS analysis for the surface molecules.

**Results:**

Cordycepin inhibited the production of NO and pro-inflammatory cytokines such as IL-1β, IL-6, and TNF-α in LPS-activated macrophages via suppressing protein expression of pro-inflammatory mediators. T2D regulating genes such as 11β-HSD1 and PPARγ were decreased as well as expression of co-stimulatory molecules such as ICAM-1 and B7-1/-2 were also decreased with the increment of its concentration. In accordance with suppressed pro-inflammatory cytokine production lead to inhibition of diabetic regulating genes in activated macrophages. Cordycepin suppressed NF-κB activation in LPS-activated macrophages.

**Conclusion:**

Based on these observations, cordycepin suppressed T2D regulating genes through the inactivation of NF-κB dependent inflammatory responses and suggesting that cordycepin will provide potential use as an immunomodulatory agent for treating immunological diseases.

## INTRODUCTION

Obesity is the most common metabolic disease in the industrial world (1). The prevalence of obesity is increasing at an alarming rate along with its associated morbidities, such as atherosclerosis and type 2 diabetes (2). Diabetes mellitus is a metabolic disorder of the endocrine system that is found in all parts of the world and is rapidly increasing in most parts of the world (3).

Type 2 diabetes mellitus, non-insulin-dependent diabetes mellitus (NIDDM), accounts for more than 90% of diabetes patients. Insulin resistance in peripheral tissues leads to compensatory hyperinsulinemia, followed by β-cell failure, leading first to prandial and later to overt fasting hyperglycemia (4).

Recent studies have indicated that obesity is associated with a chronic inflammation state, suggesting that inflammation is a potential mechanism by which obesity leads to insulin resistance (5). The authors reported that adipocytes, especially in the obese, secrete a number of pro-inflammatory cytokines, some of which have been shown to directly inhibit insulin signaling. Adipocytokines probably act through master pro-inflammatory regulators, such as those of the NF-κB and JNK/AP-1 signaling pathways to modulate the expression of genes coding for many inflammatory proteins and to alter insulin signaling.

Macrophages, which are a type of differentiated tissue cell that originate as blood monocytes, play an important role in immune and allergic reactions as well as in inflammation (6). These cells have several functions including the killing and removal of pathogenic microbes and the processing and presentation of antigens ingested by lymphocytes (7).

**Figure 1:**
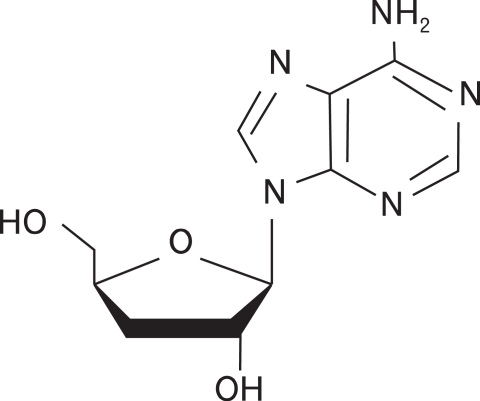
Chemical structure of cordycepin.

*Cordyceps militaris* is known as the rare Chinese caterpillar fungus (8) and has benefits to the human body including the circulatory, immune, respiratory, and glandular systems. Cordycepin (Fig. 1), 3'-deoxyadenosine, is a major component of *cordyceps millitaris* and a derivative of the nucleoside adenosine only differing from the latter by the absence of oxygen in the 3' position of its ribose entity (9). Cordycepin has been studied for anti-tumor (10), anti-inflammatory (11), anti-diabetic (12), and renoprotective effects (13). Even though cordycepin demonstrates a number of pharmacological properties, further studies are necessary to address these pharmacological differences.

The manner by which macrophages induce insulin resistance in inflammatory responses has not been established, as yet. Macrophages secrete factors induce inflammation in adipose tissue and influence insulin sensitivity, but the specific factors involved, and mechanisms by which they exert these effects, remain unknown. In this study, we tested the role of cordycepin on the NF-κB-dependent inflammation cascades and inhibition of diabetes regulating genes in lipopolysaccharide (LPS)-stimulated RAW 264.7 cells.

## MATERIALS AND METHODS

### Reagents

Cordycepin and lipopolysaccharide (LPS) were purchased from Sigma (St. Louis, USA). The cell culture media DMEM, antibiotic-penicillin/streptomycin solution and fetal bovine serum (Hyclone, Logan, USA) were used for the cell culture.

### Cell culture

Murine macrophages cell line (RAW 264.7) was obtained from the American Type Culture Collection (ATCC). Cells were cultured in Dulbecco's Modified Eagle Medium (DMEM) supplemented with high glucose, L-glutamine, 110 mg/L sodium pyruvate, 10% fetal bovine serum (FBS), and 1% (v/v) penicillin (10,000 U/ml)/ streptomycin (10,000 U/ml) (P/S). The cells were stimulated with LPS (100 ng/ml) in the presence of cordycepin for 24 hr at a concentration 2×10^5^ cells/well/200µl of media on 96-well plates for the NO assay.

### MTT assay for cell viability

A commercially-available cell viability assay was employed to evaluate the cytotoxic effect of cordycepin using thiazolyl blue tetrazolium bromide (Sigma, St. Louis, USA). RAW264.7 cells (2×10^5^ cells/well) were plated with various concentrations of cordycepin in 96-well microtiter plates (Nunc, Roskilde, Denmark) and were then cultured overnight at 37℃ in a 5% CO_2_ incubator. Afterwards, 50µl of MTT solution was added to each well, and the cells were then cultured for 4 hrs at 37℃ in a 5% CO_2_ incubator. 100µl of solubilized solution were added to each well. The plate was allowed to stand overnight in the incubator after evaluation for complete solubilization of the purple formazan crystals and the measurement of the optical density (OD) at 560 nm by a microplate reader (Molecular Devices corporation, Sunnyvale, USA).

### Measurement of NO content

To assay the total production of NO, 100µl of each culture supernatant were incubated at room temperature for 10 min with 100µl of Griess reagent (stock-I: 0.2% N-(1-naphthyl) ethylenediamine-HCl, stock-II: 2% sulfanilamide in 5% H_2_PO_4_). The O.D values of samples were read at 540 nm. A standard are curve using NaNo_2_ was then used to calculate the NO_2_^-^ concentration.

### Isolation of total RNA and RT-PCR

Total RNA was extracted from RAW 264.7 cells using the RNeasy Mini kit (QIAGEN, USA) in an RNase-free environment. RNA was quantified by reading the absorbance at 260 nm as previously described (14). The reverse transcription of 1µg RNA was carried out using M-MLV reverse transcriptase (Promega, USA), oligo (dT) 16 primer, dNTP (0.5µM) and 1 U RNase inhibitor. After incubation at 65℃ for 5 min and 37℃ for 60 min, M-MLV reverse transcriptase was inactivated by heating at 70℃ for 15 min. The polymerase chain reaction (PCR) was performed in 50 mM KCl, 10 mM Tris-HCl (pH 8.3), 1.5 mM MgCl_2_ and 2.5 mM dNTPs with 5 units of Taq DNA polymerase and 10 pM of each primer set for 11β-htdroxysteroid dehydrogenase type 1 (11β-HSD1), peroxisome proliferators-activated receptor γ (PPARγ), and regulated upon activation normal T-cell expressed and secreted (RANTES). The cDNA was amplified by 35 cycles of denaturing at 94℃ for 45 s, annealing at 62℃ for 45 s, and extension at 72℃ for 1 min. Final extension was performed at 72℃ for 5 min. The PCR products were electrophoresed on a 1.5% agarose gels and stained with ethidium bromide. The primers used were 5' CAAGGCGGGAAAGCTCATGG 3' (forward) and 5' GGAGGAGATGACGGCAATGC 3' (reverse) for 11β-HSD1, 5' ATCATCCTCACTGCAGCCGC 3' (forward) and 5' CACACTTGGCGGTTCCTTCG 3' (reverse) for RANTES, 5' GAGCCTGTGAGACCAACAGC 3' (forward) and 5' GATTCCGAAGTTGGTGGGCC 3' (reverse) for PPARγ, and 5' GTGGGCCGCCCTAGGACCAG 3' (forward) and 5' GGAGGAAGAGGATGCGGCAGT 3' (reverse) for β-actin. β-actin was used as an internal control.

### Preparation of nuclear extracts

After culture the cells were collected and washed twice with cold PBS, resuspended in hypotonic buffer (10 mM HEPES, pH 7.9, 10 mM KCl, 1.5 mM MgCl_2_, 0.2 mM PMSF, 0.5 mM DTT, 10µg/ml aportinin). After 15 min incubation on ice, the cells were lysed by the addition of 0.1% NP-40 and vigorous vortexing for 1 min. The nuclei were pelleted by centrifugation at 12,000×g for 1 min at 4℃ and resuspended in high salt buffer (20 mM HEPES, pH 7.9, 25% glycerol, 400 mM KCl, 1.5 mM MgCl_2_, 0.2 mM EDTA, 0.5 mM DTT, 1 mM NaF, 1 mM sodium orthovanadate). The supernatant fluid was stored in aliquots at -70℃.

### Western blot analysis

RAW 264.7 cells were washed with phosphate-buffered saline (PBS) and lysed by lysis buffer (1% SDS, 1.0 mM sodium vanadate, 10 mM Tris-Cl buffer, pH 7.4) for 5 min. 20µg of protein from the cell lysates was applied to 8~12% SDS-polyacrylamide gels and then transferred to nitrocellulose membranes. The membranes were blocked with 5% skim milk in PBST solution for 1 hr. They were then incubated with anti-IL-1β, anti-IL-6, anti-TNF-α, anti-i-NOS, anti-COX-2, and anti-NF-κB monoclonal antibody for 2 hrs and washed 3 times with PBST. After incubation with alkaline phosphatase-labeled secondary antibody for 2 hrs, the bands were visualized using a Western Blot Kit with alkaline phosphatase substrate (Vector, Burlingame, USA).

### Flow cytometry

RAW 264.7 cells (1×10^6^ cells/ml) were cultures in Petri-dishes. The cells were treated with various concentration of cordycepin (10, 20, 40µg/ml) in the presence of LPS (100 ng/ml). The dishes were incubated at 37℃ for 24 hrs in humidified 5% CO_2_ incubator under standard conditions. The cells washed with PBS. The washed cells blocked with staining buffer containing 10% normal mouse serum (NMS) for 20 min on ice. The blocked cells were incubated with co-stimulatory molecules such as ICAM-1, B7-1 and B7-2 antibody for 20 min on ice. The incubated cells were washed with staining buffer at 3 times. The washed cells fixed by 1% paraformaldehyde in PBS. The fixed cells were measured by flow cytometry (Beckman coulter, Brea, USA).

### Data analysis

Data are expressed as mean±standard deviation. Statistical significance between the groups was determined by paired t-test and one-way ANOVA for repeated measures. Results with p<.05 were considered statistically significant. Data were assessed using an SPSS program (version 15.0, SPSS Inc., Chicago, Illinois).

## RESULTS

### Effect of cordycepin on cell viability

To rule out the toxic effect of cordycepin, we tested its effect on the viability of RAW 264.7 by MTT assay. The exposure of cells to cordycepin at 5~40µg/ml for 24 hr showed no significant adverse effect on the cell viability versus the untreated control (data not shown).

### Reduction of NO production in LPS-stimulated RAW 264.7 by cordycepin

**Figure 2:**
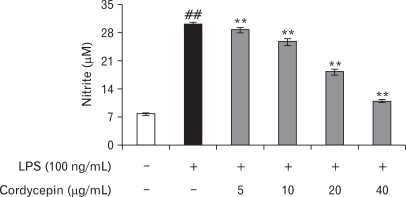
Effect of cordycepin on NO production in RAW 264.7 cells. Cells were treated with different concentrations of cordycepin; nitrite concentrations in the culture media were determined using Griess reagent assay. The results are reported as mean±S.D. of 3 independent experiments. ^##^p<0.01 vs. cells only based on Student's t-test. ^**^p<0.01 vs. cells only based on Student's t-test.

In an effort to investigate the effect of cordycepin, we first confirmed whether cordycepin inhibits NO production in activated macrophages. The macrophages did not release NO in response to the medium alone; LPS (100 ng/ml) was used as a positive control for macrophage activation (Fig. 2). When various concentration of cordycepin (5, 10, 20, 40µg/ml) were added to the culture media in the presence of LPS (100ng/ml) at the time of cell stimulation (18 hrs), NO production was decreased in a cordycepin concentration-dependent manner.

### Inhibition the gene expression of T2D regulating protein and chemokine by cordycepin

**Figure 3:**
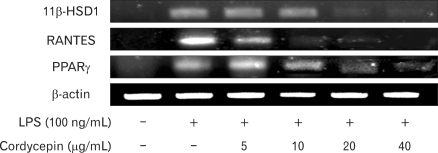
Effect of cordycepin on the expression of T2D regulating genes in RAW 264.7 cells. Levels of 11β-HSD1, RANTES, and PPARγ mRNA in RAW 264.7. Cells were incubated with various concentrations of cordycepin in the presence of LPS (100 ng/mL) for 24 hrs. The mRNA levels of T2D regulating genes were determined by RT-PCR analysis. β-actin was used as a control.

To further investigate to important role of cordycepin on T2D, murine macrophage cells with cordycepin (5~40µg/ml) in the presence of LPS (100 ng/ml) for 24 hrs decreased T2D regulating genes. As shown in Fig. 3, cordycepin suppressed 11β-HSD1, RANTES, and PPARγ expression dose-dependently.

### Effect of cordeycepin on pro-inflammatory cytokines and related proteins

**Figure 4:**
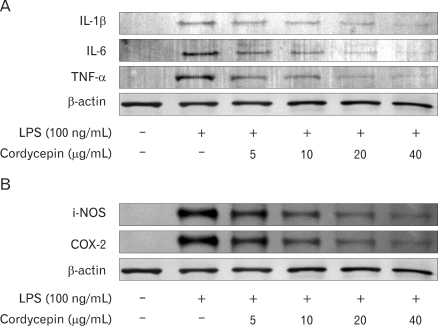
Effect of cordycepin on the expression of pro-inflammatory cytokines (A) and related proteins (B) in RAW 264.7 cells. Levels of IL-1β, IL-6, and TNF-α (A) and i-NOS and COX-2 (B) in RAW 264.7 cells. Cells were incubated with various concentrations of cordycepin in the presence of LPS (100 ng/ml) for 24 hrs. Protein (20µg) from each sample was resolved in 8~12% SDS-PAGE and then analyzed by Western blotting. β-actin was used as a control.

We determined the intracellular levels of pro-inflammatory cytokines, and related proteins by western blot analysis, showing that cordycepin decreased IL-1β, IL-6, TNF-α, i-NOS, and COX-2 in a dose-dependent manner (Fig. 4).

### Regulated the surface expression levels of co-stimulatory molecules

**Figure 5:**
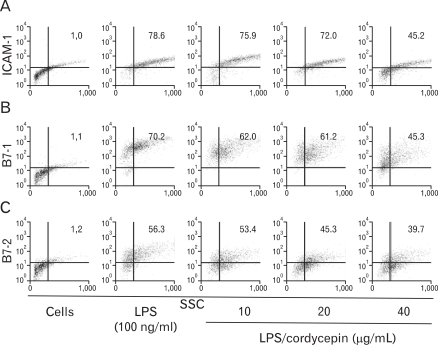
Effects of cordycepin on the expression costimulatory molecule. RAW 264.7 cells were cultured with various concentrations of cordycepin (10, 20, 40µg/ml) in the presence of LPS (100 ng/ml) for 24 hours. The surface ICAM-1 (A), B7-1 (B), and B7-2 (C) molecules were labeled with either anti-ICAM-1, anti-B7-1/-2.

The RAW 264.7 cell surface expression of ICAM-1, B7-1, and B7-2 was examined by flow cytometry. As shown in Fig. 5, cordycepin inhibited cell surface molecules such as ICAM-1, B7-1, and B7-2 in a dose-dependent manner. LPS-stimulated RAW 264.7 treated with a high concentration of cordycepin (40µg/ml) had a greater reduction than other concentration.

### Suppressed NF-κB activation by cordycepin

**Figure 6:**
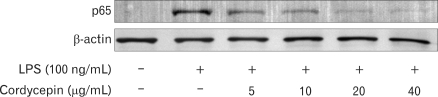
Effect of cordycepin on NF-κB activation. Levels of NF-κB protein in RAW 264.7 cells. Cells were incubated with various concentrations of cordycepin in the presence of LPS (100 ng/ml) overnight. Protein from each sample was resolved in 12% SDS-PAGE and then analyzed by Western blotting. β-actin was used in as a control.

To investigate whether cordycepin could affect nuclear translocation of NF-κB, western blot analysis for NF-κB p65 was carried out with cell lysate in macrophages (Fig. 6). Amount of NF-κB p65 was markedly increased upon exposure to LPS alone, but cordycepin decreased NF-κB p65.

## DISCUSSION

The data presented in this paper indicated that cordycepin can exert significant anti-diabetic effects on macrophage-mediated immune responses. The present study demonstrated that cordycepin suppressed NO generation, cytokine (IL-1β, IL-6, and TNF-α) expression, and co-stimulatory molecules in RAW 264.7 cells. M1 macrophages exposed to the classic activation signals IFN-γ and LPS express opsonic receptors, whereas M2 macrophages are characterized by abundant levels of non-opsonic receptors (15). Inflammation is a complex process involving numerous mediators of cellular and plasma origins. M1 macrophages fuse their lysosomes more efficiently to phagosomes, exposing intracellular or recently ingested extracellular microbes to a variety of microbiocidal lysosomal enzymes. M1 macrophages also produce oxygen radicals and NO, both of which have potent antimicrobial activity.

Arginine metabolism is characterized by high levels of iNOS in M1 macrophages, whereas the arginase pathway predominates in M2 polarized macrophages. NO is synthesized via the oxidation of arginine by a family of NOS and plays a vital role in regulating physiological processes, such as blood vessel tone and neurotransmission, as well as in host defense and immunity (16,17). However, increasing evidence indicates that NOS plays a complex role in modulating inflammatory response (18). Among these cytokines, IL-1β, IL-6, and TNF-α, have attracted more attention in that they can be localized to the infected tissue, manifested systemically throughout the body and cause vasodilation as well as symptoms of inflammation, such as redness, swelling, heat, and pain (19).

Cordycepin down-regulated the expression of pro-inflammatory molecules likewise NO in LPS-stimulated RAW 264.7 cells (Fig. 2) and when we examined the morphological changes that took place in macrophages treated with cordycepin and LPS, cells treated with LPS and low concentrations of cordycepin (5~10µg/ml) were similar those exposed to LPS alone. However, cells treated with high concentration of cordycepin (40µg/ml) in combination with LPS were smoother than those treated with LPS alone (data not shown). Also it inhibited the activation of pro-inflammatory cytokines and related proteins in both of the LPS-activated cell types (Fig. 4).

Macrophages secreted anti-inflammatory cytokines by cordycepin, it differentiate M2 macrophages. M2 macrophages are generally characterized by low production of pro-inflammatory cytokines and high expression of scavenger receptors (15). Cordycepin increased TG accumulation in macrophages (Supplementary Fig. 1). It indicated that M2 macrophages uptake TG from circulation, then are used for fuel (20).

Expression of cytokines requires the activation of NF-κB. NF-κB, a nuclear transcription factor, regulates the expression of various genes, including IL-1β, i-NOS, and COX-2 that play critical roles in apoptosis and autoimmune diseases. Its activation requires phosphorylation of IκB, by which lead IκB to ubiquitination and degradation. As shown in Fig. 6, cordycepin decreased pro-inflammatory mediators via suppression of NF-κB activation in murine macrophages.

Growing evidence has pointed to a correlative and causative relationship between inflammation and insulin resistance. The pro-inflammation cytokines such as TNF-α and IL-6 have been demonstrated to mediated insulin resistance as a result of obesity in many rodent obesity models (21,22). TNF-α was overexpressed in white adipose tissue (WAT) in obese an insulin-resistance states; mice lacking the TNF-α ligand or the p55 TNF receptor were partially protected from obesity-induced insulin resistance (23).

Increasingly, insulin resistance has been recognized as the integral feature of the so-called metabolic syndrome, which includes glucose intolerance, insulin resistance, obesity, hypertriglyceridemia, hypertension, and accelerated atherosclerosis (24).

Glucocorticoids regulate adipocyte differentiation, function and distribution, and in excess, cause visceral fat obesity and convergence of metabolic disease (25). Glucocorticoid receptor (GR) is controlled by isozymes of 11β-HSD. Although two isoforms have been identified, it is 11β-HSD1 that has attracted attention with respect to therapeutic inhibition. 11β-HSD1 is a bidirectional enzyme that resides within the endoplasmic reticulum and is widely expression in many glucocorticoid target tissues including liver, adipose tissue where it acts locally to regenerate active cortisol from inactive cortisone and thereby amplify GR activation (26).

RANTES is increased in WAT in the setting of murine and human obesity. Both mRNA and protein levels of RANTES were increased in a gender-dependent fashion in WAT of obesity. RANTES levels were particularly elevated in male mice in the stromal/vascular fraction of WAT as compared with its adipocyte fraction. In addition, monoclonal antibodies directed against RANTES reduced T-cell chemotaxis induced by media conditioned by WAT isolated from obese male mice. These findings underscore the role of RANTES-induced T-cell chemotaxis by WAT in obesity and suggest an opportunity for pharmacological interventions (27).

The transcription factor PPAR is a member of steroid receptor superfamily and has three subtypes named α, δ/β, γ (28). Specially, PPARγ is characterized originally as a key regulation of adipocyte differentiation and lipid metabolism. And it has been shown in macrophage foam cells in atherosclerotic plaques (29).

As shown in Fig. 3, cordycepin decreased diabetes regulating genes such as 11β-HSD1, RANTES, and PPARγ in activated macrophages.

IL-1 receptor antagonist (IL-1ra) is a antagonist to the pro-inflammatory cytokine IL-1 receptor, anti-inflammatory cytokine, and it is commonly thought to play an important role in the regulation of inflammatory responses, as corroborated by the enhanced sensitivity of IL-1ra knock-out mice to septic shock and their predisposition to the spontaneous development of inflammatory disorders (30,31). IL-1ra mRNA may contribute to regulation of IL-1ra synthesis that another mechanism may involve inefficient functioning of polyadenylation determinants present in the distal portion of the IL-1ra 3'-untranslated region (3'-UTR) (32).

Cordycepin is an inhibitor of transcription and polyadenylation (33), so that gene expression of IL-1ra in our data remained unprocessed (data not shown).

Intracellular adhesion molecules (ICAMs), ICAM-1, ICAM-2 and ICAM-3, are cell surface ligands for leukocyte integrins. They are crucial in the binding of lymphocytes and other leukocytes to certain cells including antigen-presenting cells (APCs). Cordycepin consistently suppressed expression of ICAM-1 surface molecule in macrophages (Fig. 5A). The B7 family plays an important role as a co-stimulatory factor in APCs. Cordycepin treatment had another major effect on co-stimulatory molecules B7-1/-2 by strongly down-regulating the surface levels of B7-1/-2 molecules in macrophage cells (Fig. 5B, C). All these molecules have been described to be of major importance in APC function (34,35).

In conclusion, we have demonstrated that cordycepin possessed anti-inflammatory and anti-diabetic effects on macrophages. Our findings are supported by inhibitory activity of cordycepin on the NF-κB pathway and other bioactive substances, as well as inhibited expression of diabetes regulating genes.
